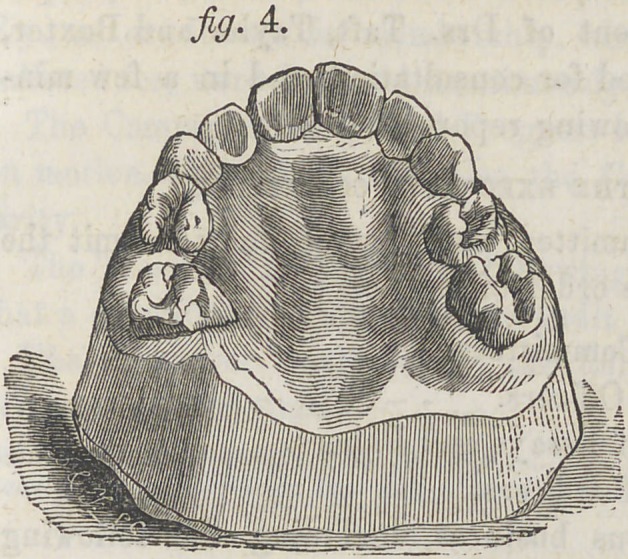# Irregularity—Treatment

**Published:** 1860-04

**Authors:** Geo. Lupton


### IRREGULARITY—TREATMENT.

Read before the Indiana State Dental Association, January, 1860.

BY DR. GEO. LUPTON.

The subject of mechanical dentistry being under consideration, Dr. Johnson moved, that the regular order of business be suspended for the purpose of giving Dr. Lupton an opportunity of presenting some models, and exhibiting a very ingenious contrivance, invented by him, for correcting irregularities of the teeth.

Dr. Lupton remarked that he was very much surprised that the committee in preparing the order of business had neglected to mention the treatment of irregularities, as he considered it a subject of vast importance, not only to the dental profession, but to the community in general; and one which had not received that attention at the hands of a majority of dental practitioners in the west that it deserved. As he did not wish to trespass on the time of the convention, he would only call their attention to one out of the number which he exhibited.

The patient, a lad of 10½ years, in which the superior central incisors fell inside of the inferior, at each occlusion of the jaws, as represented in figure 1; they also stood in a

*fig.* 1.

diagonal position, far back of, and overlaping the lateral incisors, as shown in figure 3. Recourse was had to incline1 planes, attached to a plate swedged, to fit the lower teeth, but with little benefit, as the patient soon got in the habit of letting his teeth rest on the top of the planes, as is frequently the case; consequently it was thrown aside as worthless.

*fig.* 2.

*fig.* 3.

As the permanent molars were so short as to afford no opportunity of attaching ligatures to them, it was found necessary to fit a plate to the roof of the mouth, on which to place attachments for the ends of the ligature, and having always entertained a very serious objection to the to the use of pins or standards, on account of their being in the way of the tongue, and the difficulty of tying a ligature to them when stretched to its utmost tension, he therefore adopted the following plan as being more simple and efficacious, and much more easily applied, having soldered two narrow strips to the plate (*a a fig.* 2) in the position that the standards should occupy, in such a manner as to form short, stiff springs, leaving only space enough between them and the plate to force the ligature into—the spring holding it firmly in its place without the necessity of a knot. Being very much annoyed by having to replace or tighten the ligatures every day or every few days, I at last hit upon the following plan to obviate that difficulty: finding a space on each side where teeth had been removed, I conceived the idea of inserting small, square blocks of india rubber in those spaces, and passing the ligature over them, thereby keeping up the tractile force of the ligatures for a much greater period of time; but here again I met with difficulties, being unable to keep them in their positions. I then had recourse to the following expedient,—I formed in the space where the teeth had been removed two small grooves or troughs, (*B B fig.* 2) with the posterior sides open for the reception of the blocks of india rubber; by this means they were held firmly in their places. I also formed a similar one (*B*) back of the central incisors. The ligature was then made fast to one of the springs on the palatial surface and carried out over the block of rubber on that side, passing along the labial surface of the teeth to the left lateral incisor, passing between it and the overlaping central, it was carried over the block of rubber at that point, and behind the right central; passing out between it and the right lateral, thence back to the block of rubber on that side, over which it passed and was made fast to a spring similar to the one before mentioned on the opposite side of the palatial surface, as represented in figure 2. I would here remark, however, that the blocks of rubber may be applied at such points and in such numbers as the exigencies of the case may require. I would further add, that in my opinion, there is no operation that the dentist is called upon to perform, that requires so great an amount of *determined, untiring perseverance*, as the correction of irregularities.

*fig.* 4.

In the case above alluded to the teeth were brought into place as shown in fig. 4 and the plate removed on the fifteenth day. In the above mode of applying ligatures, they do not require removal oftener than once in two weeks.

## Figures and Tables

**Fig. 1. f1:**
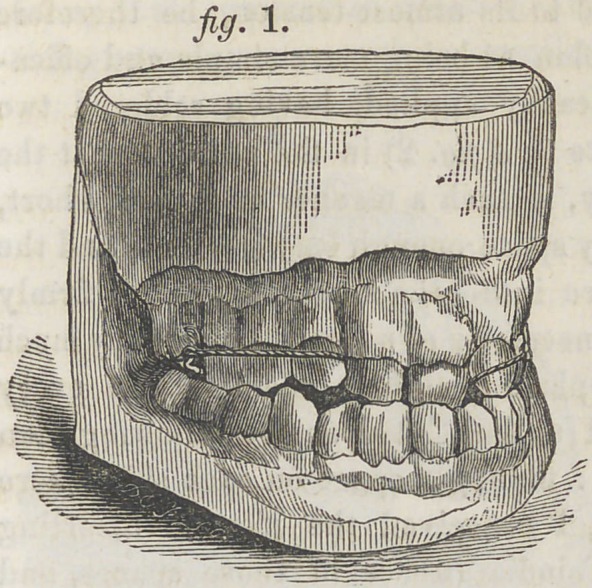


**Fig. 2. f2:**
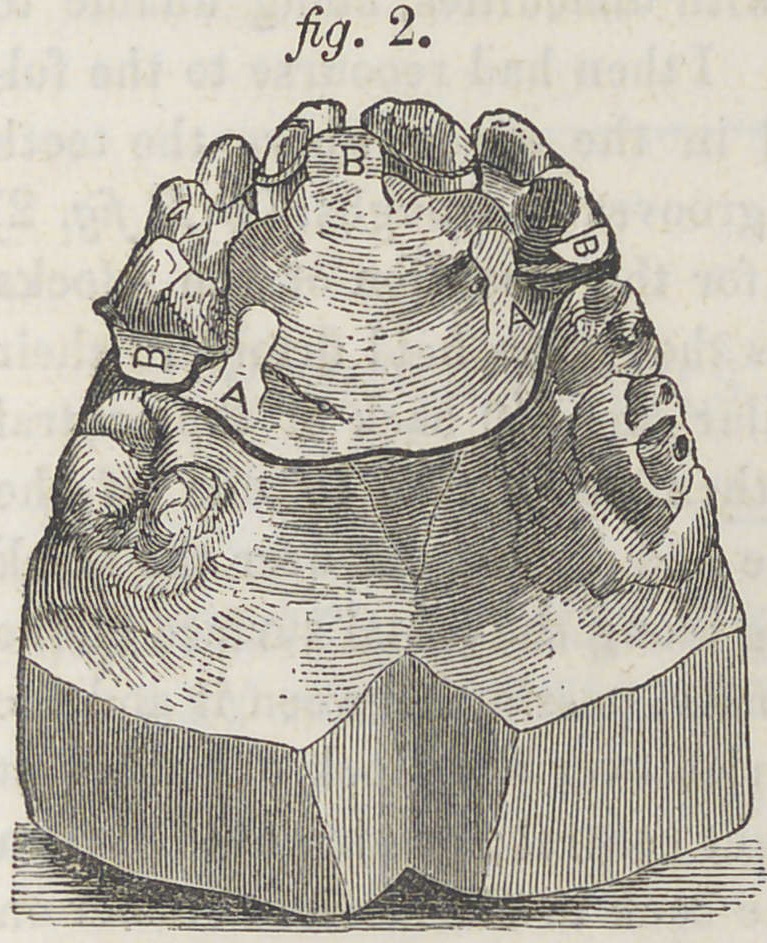


**Fig. 3. f3:**
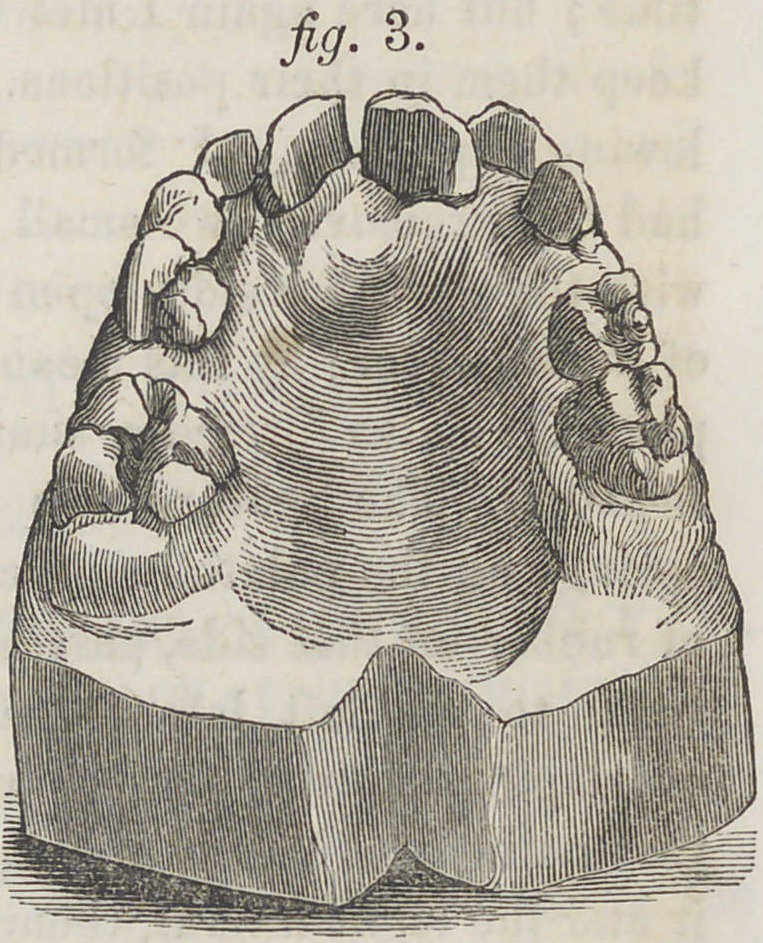


**Fig. 4. f4:**